# Acupoint application improves IVF outcomes and rescues granulosa cell steroid metabolic dysregulation in ovarian endometriosis

**DOI:** 10.3389/fendo.2025.1665669

**Published:** 2025-10-27

**Authors:** Kai-Liang Ai, Xian-Ling Cao, Yu-Qi Gao, Jing-Xian Cao, Zhen-Gao Sun

**Affiliations:** ^1^ The First Clinical College, Shandong University of Traditional Chinese Medicine, Jinan, China; ^2^ Reproductive and Genetic Center, The Affiliated Hospital of Shandong University of Traditional Chinese Medicine, Jinan, China; ^3^ Gynecology Section, Binzhou Hospital of Traditional Chinese Medicine, Binzhou, China

**Keywords:** endometriosis infertility, acupoint application therapy, *in vitro* fertilization - embryo transfer, non-targeted metabolomics, ovarian endometriosis, steroid metabolism, follicular fluid

## Abstract

**Background and objective:**

Ovarian endometriosis (OE), characterized by endometriotic cysts, adversely affects ovarian function and comprises *in vitro* fertilization and embryo transfer (IVF-ET) outcomes. Acupoint application therapy (AAT), which integrates transdermal drug delivery with acupoint stimulation, may offer therapeutic benefits; however, its underlying mechanisms remain unclear.

**Methods:**

In this randomized trial, 81 IVF-ET patients were stratified into: a treatment group comprising OE patients (n=27) undergoing a gonadotropin hormone-releasing hormone (GnRH) antagonist protocol with medicated AAT, a placebo group consisting of OE patients (n=26) following an identical protocol but receiving a sham patch, and a control group, including patients with male-factor infertility (n=28) undergoing the standard protocol without additional interventions. Follicular fluid metabolomics (assessed by Ultra High-Performance Liquid Chromatography-Mass Spectrometry (UPLC-MS/MS) System)and IVF parameters were analyzed.

**Results:**

Significant intergroup differences were observed (one-way ANOVA, *P* < 0.05). The treatment group exhibited a shorter Gn duration (9.00 ± 0.68 days) compared to the placebo group (10.62 ± 2.43 days; *P* < 0.05), with a duration comparable to the male-factor control group (9.32 ± 1.89 days; NS). Similarly, the total Gn dose was lower in the treatment group (2112.50 ± 483.17 IU) than in the placebo group (2549.04 ± 677.44 IU; *P* < 0.05), and comparable to the control group (2105.89 ± 690.24 IU; NS). Regarding IVF outcomes, the treatment group yielded more oocytes retrieved (12.41 ± 7.27) than the placebo group (8.85 ± 7.89; *P* < 0.05), though fewer than the control group (15.25 ± 7.77). Fertilized oocytes were also higher in the treatment group (7.59 ± 4.58) compared to the placebo group (4.46 ± 3.40; *P* < 0.05), but fewer than in the control group (9.21 ± 4.82). The number of transferable embryos was comparable between the treatment group (3.33 ± 2.30) and the control group (3.43 ± 2.13; NS), and significantly higher than in the placebo group (1.69 ± 1.87; *P* < 0.05). Furthermore, the treatment group produced more high-quality embryos (3.04 ± 1.89) than the placebo group (1.35 ± 1.99; *P* < 0.05), and more than the control group (2.43 ± 1.95). No significant intergroup difference was found in fertilization rates (64.40% vs. 62.90% vs. 60.90%; NS). However, the high-quality embryo rate was significantly higher in the treatment group (47.02%) compared to both the placebo (31.15%; *P* < 0.05) and control (29.05%; *P* < 0.05) groups. Finally, the treatment group demonstrated a significantly greater reduction in peri-menstrual abdominal pain scores (Δ=−1.15 ± 0.36) compared to the placebo group (Δ=−0.35 ± 0.49; F = 6.84, *P* = 0.01). Metabolomic analysis revealed that the steroid hormone biosynthesis pathway was the most significantly disturbed in OE patients, characterized by markedly elevated levels of key intermediates such as 17α-hydroxyprogesterne. Critically, the levels of 17α-hydroxyprogesterone exhibited a significant negative correlation with oocyte yield (r = -0.286, p = 0.012), directly linking this metabolic dysregulation to impaired clinical outcome. Furthermore, oxidative stress metabolites showed a strong positive correlation with the luteinization marker 20α-hydroxy-4-pregnen-3-one (r = 0.43, p = 0.001), suggesting a potential interaction between oxidative stress and aberrant steroidogenesis. The AAT intervention effectively normalized this dysregulated steroidogenic profile, which underpinned the observed therapeutic benefits.

**Conclusion:**

AAT significantly alleviates peri-menstrual pain and enhances IVF outcomes in ovarian endometriosis patients, which may be attributed to the restoration of granulosa cell steroid metabolism, evidenced by normalized levels of 17α-hydroxyprogesterone and attenuation of oxidative stress.

**Clinical trial registration:**

https://www.chictr.org.cn/, identifier ChiCTR2200057339.

## 1 Introduction

Ovarian endometriosis (OE), characterized by the formation of endometriotic cysts (“chocolate cysts”), represents a particularly severe subtype of endometriosis that profoundly impacts fertility. Unlike other forms (1), OE directly damages the ovarian parenchyma, leading to impaired folliculogenesis, reduced oocyte quality, and diminished ovarian reserve (2, 3). These pathological changes, driven by a dysfunctional microenvironment marked by chronic inflammation, oxidative stress, and aberrant steroid metabolism, contribute to poor *in vitro* fertilization (IVF) outcomes, including reduced ovarian response and higher cycle cancellation rates.

International guidelines consider minimally invasive surgery the primary approach for OE patients seeking conception; however, surgery may further compromise ovarian function (3). Current non-surgical treatment options include: 1) Hormonal therapies, such as GnRH agonists (4), which induce down-regulation to suppress endometriotic cysts. These treatments require a significant time commitment (3–6 months), during which patients face the risk of declining ovarian reserve and must endure side effects including emotional fluctuations, potentially exacerbating anxiety and even contributing to familial discord (5). 2) Non-steroidal anti-inflammatory drugs (NSAIDs), which are first-line agents for dysmenorrhea but do not alter the pathological state of OE and thus offer no benefit for improving assisted reproductive outcomes, Critically, none of these approaches adequately address the dysregulated ovarian microenvironment, highlighting an urgent need for novel adjuvant therapies that can improve IVF outcomes without iatrogenic harm.

Traditional Chinese Medicine (TCM) has emerged as a promising complementary paradigm for complex chronic conditions like endometriosis, owing to its holistic regatory philosophy, multitarget mechanisms, and emphasis on personalized treatment (5–10). Acupoint application therapy (AAT)—a non-invasive modality that combines transdermal drug delivery with sustained acupoint stimulation—has demonstrated anti-inflammatory, antioxidant, and endocrine-modulating effects in conditions including Hashimoto’s thyroiditis, asthma, and allergic rhinitis. Notably, Zhang et al. reported that AAT can regulate estrogen receptor expression in gynecological tissues, suggesting its potential to interact with estrogen-dependent pathologies such as OE (11). For OE patients undergoing IVF, AAT offers several distinct clinical advantages: 1) its non-invasive nature avoids further damage to ovarian tissue; 2) it can be seamlessly integrated into IVF cycles without prolonging treatment time or disrupting protocols; 3) it exerts multitarget effects, potentially modulating oxidative stres (12, 13), chronic inflammation (14, 15), and steroid metabolism (11, 16)—core aspects of OE pathogenesis; and (4) it may confer additional benefits such as pain reduction and emotional relief (17, 18).

We specifically focused on granulosa cell steroid metabolic dysregulation due to its central role in folliculogenesis, oocyte maturation, and steroid hormone biosynthesis. Clinically, steroid levels are routinely used as key indicators of follicular quality. In OE, steroid metabolic dysregulation—characterized by aberrant estrogen signaling and progesterone resistance—represents a core pathological feature (19). As the primary source of ovarian steroidogenesis, dysfunctional granulosa cells constitute a critical mechanistic link between OE pathology and associated infertility (20). Furthermore, evidence indicates that steroid metabolic abnormalities, oxidative stress, and chronic inflammatory responses are closely interrelated in endometriosis, forming a self-sustaining vicious cycle that exacerbates ovarian dysfunction (21). Therefore, we considered that targeting steroid metabolism could serve as a pivotal therapeutic strategy for interrupting this pathogenic network. We hypothesized that AAT may ameliorate OE-associated follicular dysfunction primarily by restoring steroidogenic homeostasis in granulosa cells, with potential additional effects on associated inflammatory and oxidative pathways. Thus, this study aimed to evaluate the effects of AAT on IVF outcomes and to investigate its underlying mechanisms using non-targeted metabolomics, with a particular emphasis on granulosa cell-centered steroid metabolism.

## 2 Participants and methods

### 2.1 Patients and inclusion/exclusion criteria

This multicenter prospective study recruited patients with ovarian endometriosis from four hospitals in Shandong province between March 2022 and September 2022, including the Affiliated Hospital of Shandong University of Traditional Chinese Medicine (Jinan), Jinan Integrated Chinese and Western Medicine Hospital, Zibo Traditional Chinese Medicine Hospital, and Yantai Mountain Hospital (Yantai).

The inclusion criteria were as follows: 1. A diagnosis of endometriosis primarily confirmed by ultrasound images showing cystic lesions with ground-glass echoes around the ovary on ultrasound images (22); 2. Aged 18–45 years with regular menstrual cycles (21–35 days), menstruation duration of 3–7 days, and scheduled for IVF-ET treatment; 3. Patients with ovarian mass diameter < 4 cm to mitigate the risk of cyst rupture or torsion during controlled ovarian stimulation, a process associated with significant ovarian enlargement and increased vascular permeability; 4. Serum CA125 levels between 35IU/L and 80IU/L; 5. Provision of written informed consent. The blank control group comprised female infertile patients who underwent assisted reproductive IVF due to male factor infertility.

The exclusion criteria were as follows: 1. Dysmenorrhea caused by primary dysmenorrhea or other underlying diseases; 2. Participation in other new drug clinical experiments within the past 3 months; 3. Diagnosed coagulation disorder; 4. Documented allergies to multiple drugs or a generalized allergic diathesis; 5. Concomitant mental illness, alcoholism, and/or psychoactive substance abuse and dependence; 6. Patients with skin trauma, ulcers, skin infection, or scar at the application site and a known allergy to the patch material; 7. Pregnancy or lactation.

A blank control group was additionally established, which was not generated from the randomization sequence. This group consisted of prospectively recruited female patients undergoing IVF-ET solely due to male factor infertility (e.g., oligoasthenospermia, obstructive azoospermia), who were rigorously screened to confirm the absence of female infertility factors. Participants in this group were required to meet key criteria—including age matching (18–45 years), normal ovarian function (regular menstrual cycles, normal baseline hormone levels, and normal antral follicle count), absence of pelvic pathology (confirmed by transvaginal ultrasound), and identical GnRH antagonist treatment protocol—to ensure maximal comparability with the OE trial groups. The purpose of this group was to establish a disease-free baseline for ovarian response and embryonic development, thereby enabling a clearer assessment of both the impact of ovarian endometriosis itself and the specific effect of AAT intervention on IVF outcomes.

### 2.2 Acupoint selection, herbal formulation, and application protocol

(1) Treatment Group: The acupoints selected for the treatment group were Yongquan (KI1, bilateral), Yashi (bilateral), and Shenque (CV8). The following Chinese herbal formulations were applied: 1. At bilateral Yongquan (KI1): Evodia rutaecarpa (0.25g) and borneol (0.1g); 2. At bilateral Yashi: Sinapis alba (0.25g) and Asarum sieboldii (0.25g); 3. At Shenque (CV8): Zingiber officinale (dried ginger, 0.25g) and Chuanxiong rhizoma (0.25g). The fine powder of the herbs was provided by Yabao Pharmaceutical Group Co., Ltd. The external application carrier was the Xiaozhong Zhitong Plaster (Specifications: each medicated patch weighs 0.4g; The liquid medicine, provided in 2ml per tube, contains *Artemisia desertorum* menthol, and *Sisymbrium officinale* as its active ingredients.), also manufactured by Yabao Pharmaceutical Group Co., Ltd.
(2) Usage: The Yeshi acupoints (bilateral) received application once weekly for 30 minutes per session. The Shenque acupoint was treated once daily for 6–8 hours per application. The Yongquan acupoints (bilateral) were also treated once daily for 6–8 hours per application.
(3) Selection of acupoints and medication for the placebo group:The placebo was manufactured by the Shandong Academy of Chinese Medicine and primarily consisted of starch and non-pharmacologically active flavorings, designed to mimic the appearance, color, and smell of the active treatment. The selection of acupuncture points and the application protocol for the placebo group were identical to those used in the treatment group.

### 2.3 Clinical pharmacological agents

(1) r-FSHβ (Recombinant Follitropin Beta, Puregon^®^), Merck Serono SA Aubonne Branch, S20150065, 600IU/vial;
(2) HMG (Human Menopausal Gonadotropin), Livzon Baio Biological Pharmacy, Guoyao Zhunzi H10940097, 75IU/vial;
(3) GnRH-A (Cetrorelix Acetate, Cetrotide^®^), Baxter Oncology GmbH, H20100369, 0.25mg/vial;
(4) HCG (Human Chorionic Gonadotropin), Livzon Baio Biological Pharmacy, Guoyao Zhunzi H44020674, 2000IU/vial;
(5) r-HCG (Recombinant Human Chorionic Gonadotropin, Ovidrel^®^), Serono Europe Ltd., S20050093, 250μg/vial.

### 2.4 Study design

#### 2.4.1 Sample size calculation

The sample size was calculated *a priori* using PASS software (version 15.0, NCSS, LLC) based on the primary outcome of the total number of oocytes retrieved, specifically for the comparison between the AAT and placebo groups. Based on historical data from antagonist cycles at our reproductive center and previous literature (16), we assumed a mean ± SD of 10 ± 4 oocytes for the AAT group and 7 ± 4 for the placebo group. With a two-sided significance level (α) of 0.05 and a statistical power (1-β) of 80%, a minimum of 29 patients per group was required to detect this mean difference of 3.Although the calculated sample size was 29 per group, we ultimately enrolled 27 patients in the AAT group and 26 in the placebo group due to challenges in patient recruitment during the study period. *Post hoc* power analysis indicated that the achieved sample size provided 78% statistical power to detect the prespecified mean difference of 3 oocytes, which we consider acceptable for this preliminary investigation.

#### 2.4.2 Randomization method

The randomization procedure was performed using stratified block randomization with a fixed block size of 4. Participating center was used as the sole stratification factor to ensure balance between the intervention and placebo groups within each of the four clinical sites. An independent statistician, who had no contact with participants, clinicians, or outcome assessors, generated the master randomization list. For each center, a separate, unique random sequence was computer-generated using the block randomization module in SPSS software (version 26.0, IBM Corp.). To implement allocation concealment across centers, the following rigorous manual procedure was employed: 1. The independent statistician sealed the allocation sequence for each center in a set of sequentially numbered, opaque, tamper-evident envelopes. 2. Each envelope corresponded to a single participant ID number. 3. These sealed envelope sets were then distributed to the principal investigator at each respective clinical site. 4. Upon enrollment of an eligible participant, the site investigator would open the next consecutively numbered envelope in the presence of a witness to reveal the group assignment (AAT or placebo). This manual system, meticulously designed and executed, ensured that the random sequence was unpredictable and inaccessible to all personnel involved in patient recruitment and care, thereby robustly maintaining allocation concealment throughout the trial.

#### 2.4.3 Participant allocation

This prospective, randomized, double-blind, placebo-controlled trial enrolled 53 ovarian endometriosis infertility patients randomly allocated to either the treatment group (n=27), which received a GnRH antagonist protocol combined with acupoint application therapy, or the placebo group (n=26), which received an identical protocol with sham patches. Besides, a male-factor infertility control group (n=28) was concurrently enrolled, receiving the GnRH antagonist protocol only.

#### 2.4.4 Controlled superovulation protocol

All three groups underwent ovarian stimulation using the GnRH antagonist protocol. On cycle days 2-3, baseline Follicle-Stimulating Hormone (FSH), Luteinizing Hormone (LH), Estradiol (E2), and Progesterone (P) levels were measured, along with a transvaginal ultrasound, to assess ovarian reserve. Gn starting doses (150–250 IU/day) were determined by age, BMI, and baseline markers. From days 6–8 of stimulation, GnRH antagonists were initiated when dominant follicles exceeded 14mm in diameter, or E2 levels ≥400 pg/mL, or LH ≥10 IU/L. Ovulation was triggered with 10,000 IU of HCG when 2–3 lead follicles reached a diameter of 18mm, followed by ultrasound-guided transvaginal oocyte retrieval 35–36 hours later.

#### 2.4.5 Acupoint application therapy protocol

The treatment group received medicated acupoint patches for 7 days pre-menstruation and 7 days post-menstruation (total 14 days). The placebo group received identical sham patches during the same pre-/post-menstrual phases. The male-factor control group received no acupoint intervention.

### 2.5 Data collection, management, and statistical analysis

#### 2.5.1 Statistical analysis

Statistical analyses were performed using SPSS 25.0 (IBM Corp., USA). Continuous variables were presented as mean ± SD when normally distributed (assessed by the Shapiro-Wilk test) and compared using independent t-tests or one-way ANOVA with LSD *post-hoc* for homogeneous variances. Non-normally distributed data were expressed as median (IQR) and analyzed with Mann-Whitney U or Kruskal-Wallis tests. Categorical variables were reported as proportions (%) and compared by χ² or Fisher’s exact tests. For multi-group comparisons with heterogeneous variances (Levene’s test, *P* < 0.05), Welch’s ANOVA with Games-Howell *post-hoc* was applied. Statistical significance was defined as *P* < 0.05 (two-tailed).

#### 2.5.2 Experimental sample information and collection

The change in peri-menstrual abdominal pain severity was assessed via the Cox Menstrual Symptom Scale (CMSS). This self-administered scale was completed at baseline (pre-intervention) and after the 14-day intervention. The change in pain score (Δ) was derived as [post-intervention score] minus [baseline score], with negative values indicating an improvement in pain.

#### 2.5.3 Metabolomic profiling analysis

##### 2.5.3.1 Sample source and selection criteria

Follicular fluid samples for metabolomic analysis were directly derived from the pre-established clinical cohort of 81 patients enrolled in the main intervention trial. No additional selection criteria were applied, ensuring that the metabolomic subset was fully representative of the original randomized population. Participants had already been assigned to the treatment (acupoint application therapy, n=27), placebo (sham acupoint application, n=26), or blank control (n=28) groups according to the original randomization protocol.

Follicular fluid samples were collected from uncontaminated, mature follicles during oocyte retrieval. The samples were centrifuged at room temperature (3000 × g, 15 min), and the resulting supernatant was aliquoted into 5 mL EP tubes. All samples were immediately stored at −80 °C under strict protocols to prevent freeze-thaw cycles and preserve biomolecular integrity. Detailed clinical and demographic characteristics of the participants are provided in **Supplementary Table S1**.

##### 2.5.3.2 Sample preparation

All samples were melted at 4 °C. A 100 uL aliquot of each follicular fluid sample was weighed precisely in a 1.5 mL centrifuge tube. To this, 300 uL of methanol (containing internal standard 100 ng/mL chloramphenicol and clenbuterol) was added, vortexed for 5 min, and then centrifuged at 14,000 g for 30 min at 4 °C. The supernatant was aspirated into an EP tube, and 10 uL of the supernatant was injected into the sample for analysis. 10 uL of each sample was mixed into a QC sample, which was used for method optimization and to assess the reliability of the experimental results.

##### 2.5.3.3 Chromatography-mass spectrometry

###### 2.5.3.3.1 Experimental apparatus and reagents

Instruments: The analysis was performed using a UPLC-MS/MS system, including the ExionLC AD system (AB SCIEX, CA, USA) coupled with the X500R QTOF mass detector (AB SCIEX, CA, USA). Sample preparation was aided by a refrigerated centrifuge H1650-W (Eppendorf) and a vortex shaker QL-866 (Vortex Mixer).

Reagents: chromatographically pure reagents included acetonitrile (Merck, USA, batch no. 10900830); chromatographically pure methanol (Merck, USA, batch no. 10899607729), formic acid (CNW technologies GmbH, batch no. H6070170), and ammonium formate (CNW technologies GmbH, batch no. 0000195636). Pure water for analysis was generated using a GT-30L Reverse Osmosis Ultrapure Water Machine (Shanghai Feiwu Laboratory Equipment Co., Ltd).

###### 2.5.3.3.2 Chromatographic conditions

The chromatographic column was performed using a Waters T3 column (100* 2.1mm, 1.7um). For positive ion mode, mobile phase A consisted of an aqueous phase of 0.1% formic acid, while mobile phase B was acetonitrile. The flow rate was set to 0.4 mL/min, and the column temperature was kept at 40°C (**Supplementary Table S2**).

###### 2.5.3.3.3 Mass spectrometry conditions

The mass spectrometry was performed using an AB SCIEX X500R QTOF, acquiring data in positive and negative ion modes, respectively. The scanning mode was set to classical data-dependent scanning (IDA), where a single primary mass spectrometry scan (100ms) triggered 10 secondary mass spectrometry scans. In addition, dynamic background subtraction (DBS) was enabled. The primary and secondary scanning ranges were both set from 50 m/z to 1000 m/z. Instrument parameters included: air curtain gas: 35 psi; nebulization gas: 60 psi; auxiliary nebulization gas: 60 psi; ion source temperature: 550 °C; spray voltage 5500 V(+)/-4500 V (–); de-cluster voltage 60 V(+)/-60 V (–); collision Voltage 35 ± 15V(+)/-35 ± 15V (–).

##### 2.5.3.4 Total ion flow chromatogram

**Figure 1:**
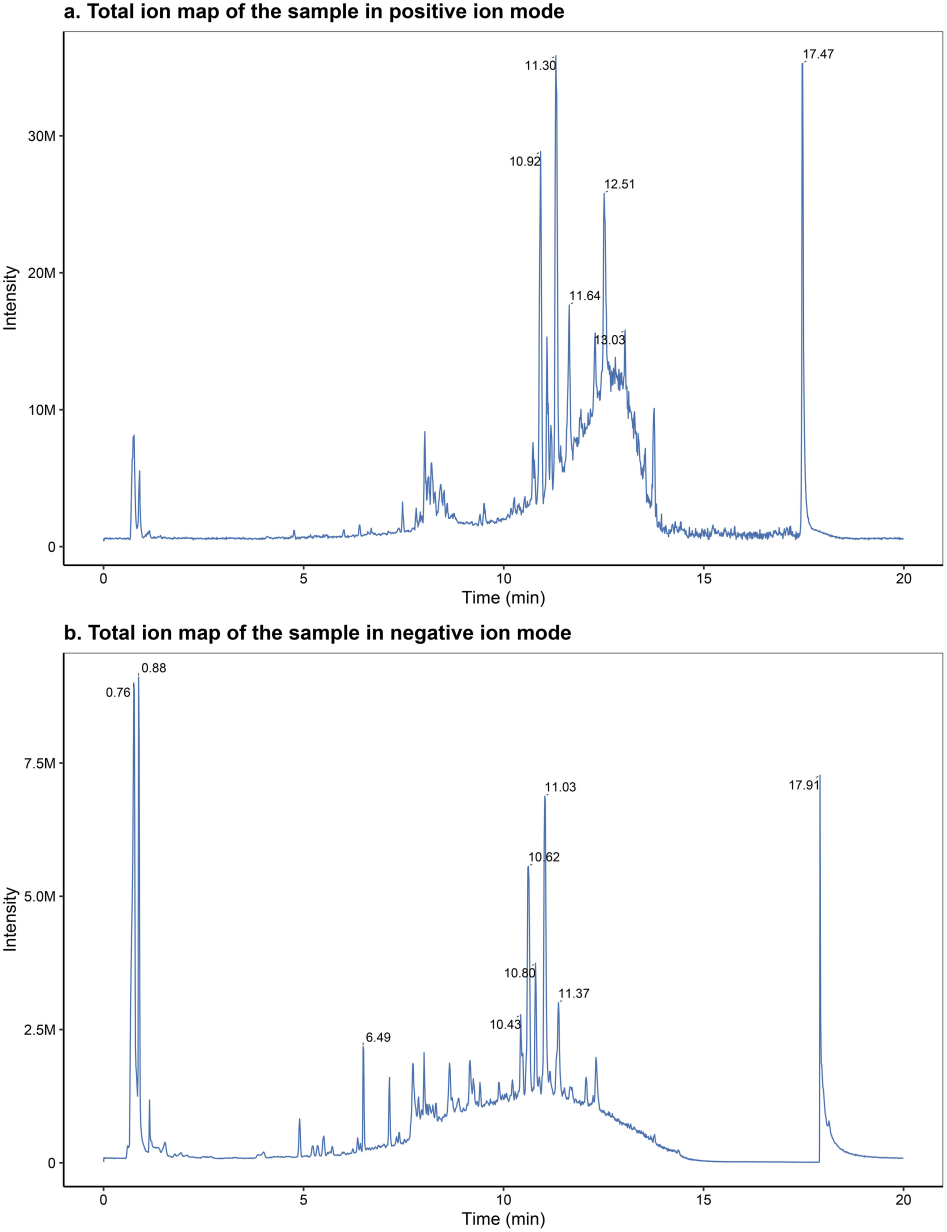
Representative total ion chromatograms (TICs) acquired in positive **(A)** and negative **(B)** electrospray ionization modes.

The chromatographically separated components were continuously introduced into the mass spectrometer, which performed continuous scanning and data acquisition. The chromatograms obtained from each scan were superimposed, with ion intensity represented on the vertical axis and retention time on the horizontal axis. The total ion flow chromatograms were obtained, as shown in **Figure 1**, panels A and B.

##### 2.5.3.5 Quality control and quality assurance

Quality control (QC): To ensure the reliability and accuracy of metabolomic data generated through liquid chromatography-mass spectrometry (LC-MS), stringent quality control measures were implemented. QC samples were integrated throughout the analytical process to monitor system stability and reproducibility. Ideally, QC samples should produce identical results; however, minor variations may arise due to systematic errors during sample extraction and analysis. The degree of variation among QC samples serves as an indicator of methodological consistency, with minor deviations reflecting greater analytical stability and higher data quality.

Quality assurance (QA): To detect biomarkers, the relative standard deviation (RSD), i.e., the coefficient of variation (CoV), of potential characteristic peaks in the QC samples should not exceed 30%. The relevant characteristic peaks exceeding this threshold should be eliminated. Therefore, poorly reproducible characteristic peaks are removed from QC samples based on quality control, resulting in high-quality datasets suitable for biomarker detection. In this experiment, the QC samples were well clustered on the score plot, and the relative standard deviation of the peak areas of the internal standards chloramphenicol and clenbuterol was less than 5%, which indicated the good reproducibility of the assay method.

##### 2.5.3.6 Data analysis and processing

**Figure 2:**
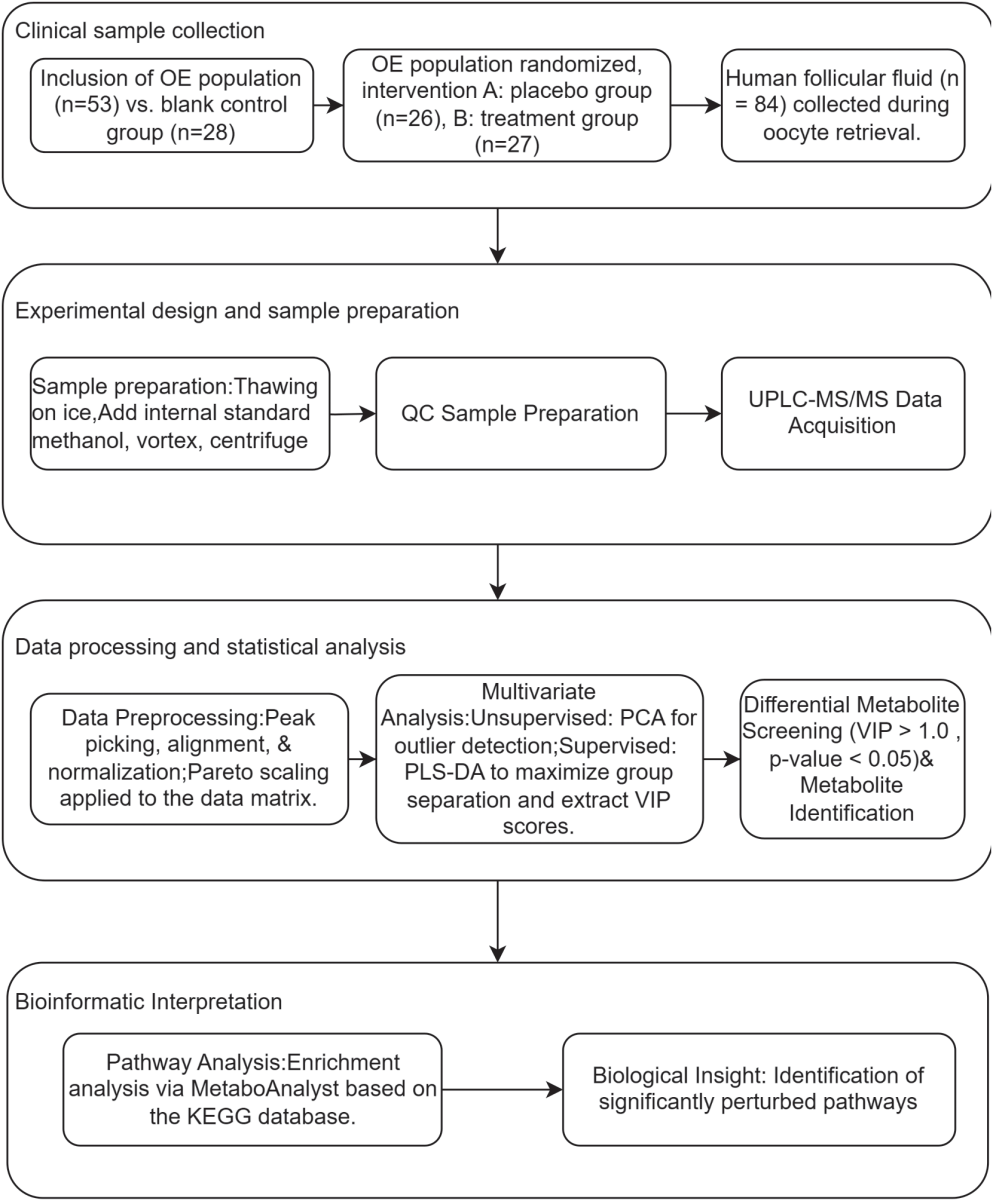
Schematic workflow of the non-targeted metabolomics analysis.

After rigorous quality control, the raw mass spectrometry data underwent a comprehensive data processing and statistical analysis pipeline, as illustrated in the research flowchart (**Figure 2**). The process commenced with data preprocessing: raw data files were imported into MS-DIAL for peak picking, alignment, and normalization, followed by Pareto scaling (Mean-centering and scaled to Pareto variance) to minimize the influence of high-abundance metabolites and heteroscedasticity. Subsequently, multivariate statistical analysis was performed. This included an unsupervised Principal Component Analysis (PCA) to assess intrinsic data clustering and identify potential outliers, followed by a supervised Partial Least Squares-Discriminant Analysis (PLS-DA) to maximize group separation and identify metabolites most responsible for the discrimination (Variable Importance in Projection, VIP > 1.0). The robustness of the PLS-DA model was validated by permutation test. For differential metabolite screening, metabolites with a VIP score > 1.0 from the PLS-DA model were further subjected to univariate analysis (Student’s t-test), and those with an FDR-adjusted p-value < 0.05 were considered statistically significant. Finally, these significantly altered metabolites were used for hierarchical clustering analysis (results presented as a heatmap) and pathway enrichment analysis based on the KEGG database to elucidate the biological implications.

## 3 Results

### 3.1 Patient information

No serious adverse events occurred across groups. Baseline characteristics, including age, infertility duration, BMI, and basal hormones (FSH, LH, E2, P), remained comparable (*F* = 0.03-2.77, *P >*0.05), while parity (*F* = 3.87, *P* < 0.05) and infertility types (χ²=7.62, *P* < 0.05) exhibited significant differences, as shown in **Table 1**.

**Table 1:** Comparison of basic information of the three groups of patients [(x̄ ± s), %].

Basic information	Treatment group (n=27)	Placebo group (n=26)	No-treatment group (n=28)	*F*	*P*
Age (years)	31.79 ± 3.87	31.50 ± 6.44	30.46 ± 5.16	0.51	0.61
Years of infertility (years) Number of pregnancies Number of times	3.34 ± 2.35 0.59 ± 0.83 0.10 ± 0.31	3.77 ± 3.78 0.96 ± 1.11 0.27 ± 0.53	4.32 ± 3.060 0.29 ± 0.71 0.14 ± 0.36	0.72 3.87 1.22	0.49 0.03 0.30
BMI (Kg/m^2^)	22.82 ± 2.65	23.41 ± 3.57	23.22 ± 4.15	0.21	0.81
Basic FSH (IU/L)	7.68 ± 3.40	8.41 ± 3.43	6.54 ± 1.69	2.77	0.07
Basic LH (IU/L)	4.43 ± 2.72	4.74 ± 2.20	5.10 ± 5.41	0.23	0.80
Basic E2 (pg/mL)	40.31 ± 13.81	41.20 ± 18.10	41.88 ± 35.90	0.03	0.97
Basic P (ng/mL)	0.59 ± 0.32	0.91 ± 0.82	1.25 ± 3.51	0.72	0.49
Primary infertility (%)	63.00 (17/27)	46.20 (12/26)	82.10 (23/28)	*χ^2^* =7.62	*P*=0.02
Secondary infertility (%)	37.00 (10/27)	53.80 (14/26)	17.90 (5/28)		

### 3.2 Clinical outcomes

Regarding ovarian stimulation outcomes, the following findings were observed (1): The total Gn dose differed significantly between the placebo group versus both the treatment and control groups (F = 4.38, *P=*0.01) (2). Gn duration was shorter in the treatment group (9.00 ± 0.68 days) compared to the placebo group (10.62 ± 2.43 days; *P=*0.012), while the placebo and control groups (9.32 ± 1.89 days) exhibited comparable durations (3). The number of oocytes retrieved in the placebo group (8.85 ± 7.89) was fewer than in the control group (15.25 ± 7.77;*P* = 0.012) but similar to the treatment group (12.41 ± 7.27; NS) (4). Fertilized oocytes(*P* = 0.001), transferable embryos(*P* = 0.005), and high-quality embryos (*P* = 0.008) were significantly reduced in the placebo group compared to both the treatment and control groups (5). Fertilization rates remained comparable across groups (64.40% vs. 62.90% vs. 60.90%; *P* = 0.812) (6). The control group exhibited the lowest high-quality embryo rate (29.05%) compared to the treatment group (47.02%; *P* = 0.023). Furthermore, the treatment group demonstrated a significantly greater reduction in peri-menstrual abdominal pain scores (Δ=−1.15 ± 0.36) compared to the placebo group (Δ=−0.35 ± 0.49; F = 6.84, *P* = 0.01). Detailed results are provided in **Table 2** and **Supplementary Table S3**.

**Table 2:** Ovulation promotion and embryo formation in three groups of patients [(x̄ ± s), %].

Clinical information	Treatment group (n=27)	Placebo group (n=26)	No-treatment group (n=28)	*F*	*P*
Gn duration (days)	9.00 ± 0.68	10.62 ± 2.43	9.32 ± 1.89	5.88	0.004
Total Gn use (IU)	2112.50 ± 483.17	2549.04 ± 677.44	2105.89 ± 690.24	4.38	0.016
Total number of eggs	12.41 ± 7.27	8.85 ± 7.89	15.25 ± 7.77	4.68	0.012
Total number of normally fertilized eggs	7.59 ± 4.58	4.46 ± 3.40	9.21 ± 4.82	8.31	0.001
Number of embryos available for transfer	3.33 ± 2.30	1.69 ± 1.87	3.43 ± 2.13	5.65	0.005
Number of high-quality embryos	3.04 ± 1.89	1.35 ± 1.99	2.43 ± 1.95	5.11	0.008
				*χ^2^*	*P*
Normal fertilization rate	64.40 (205/336)	62.90 (116/230)	60.90 (258/427)	0.209	0.812
High-quality embryo rate	47.02 (82/205)	31.15 (35/116)	29.05 (68/258)	3.78	0.023

### 3.3 Analysis of metabolites

**Figure 3:**
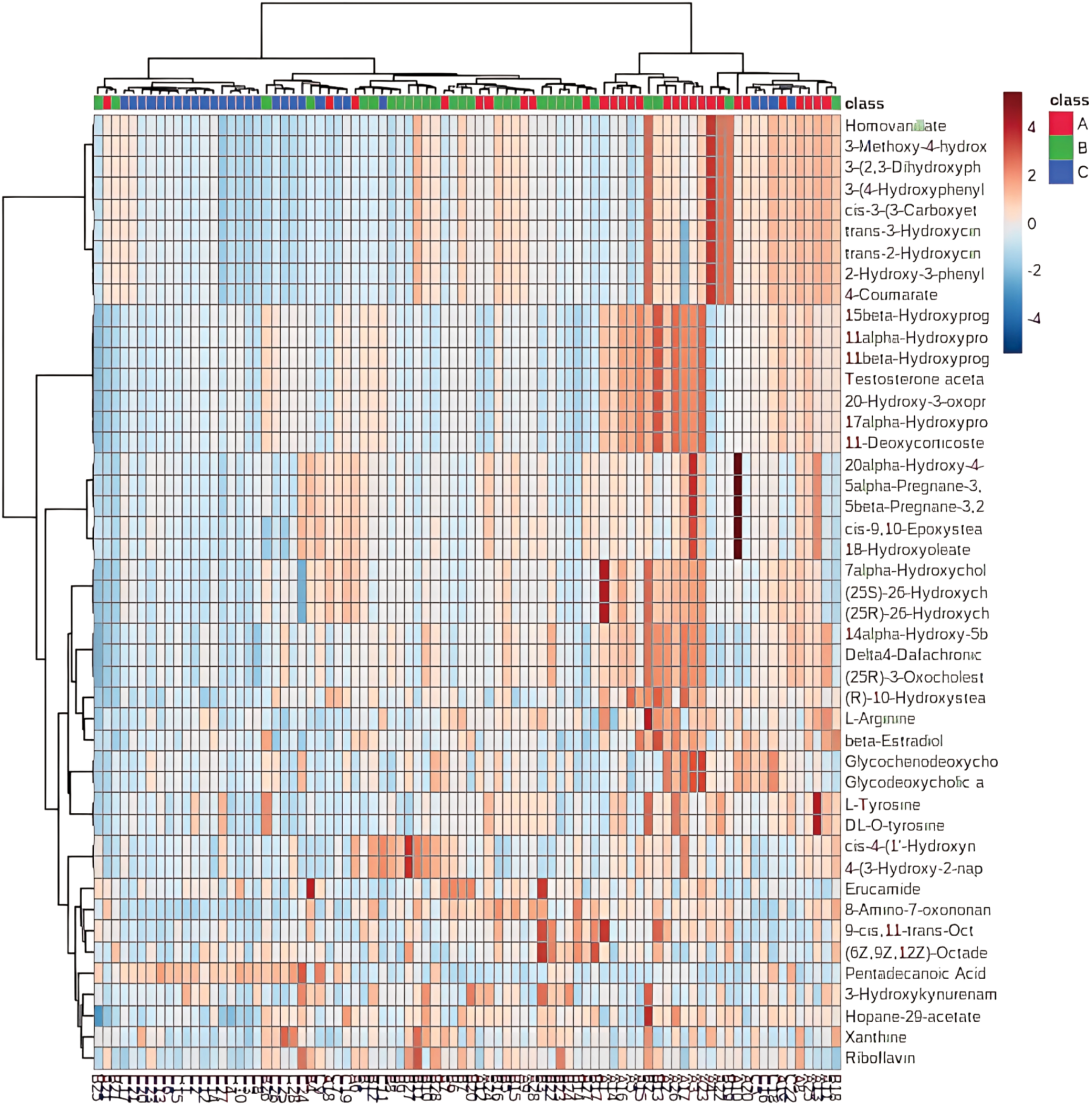
Hierarchical clustering analysis of metabolite profiles across study groups.

**Figure 4:**
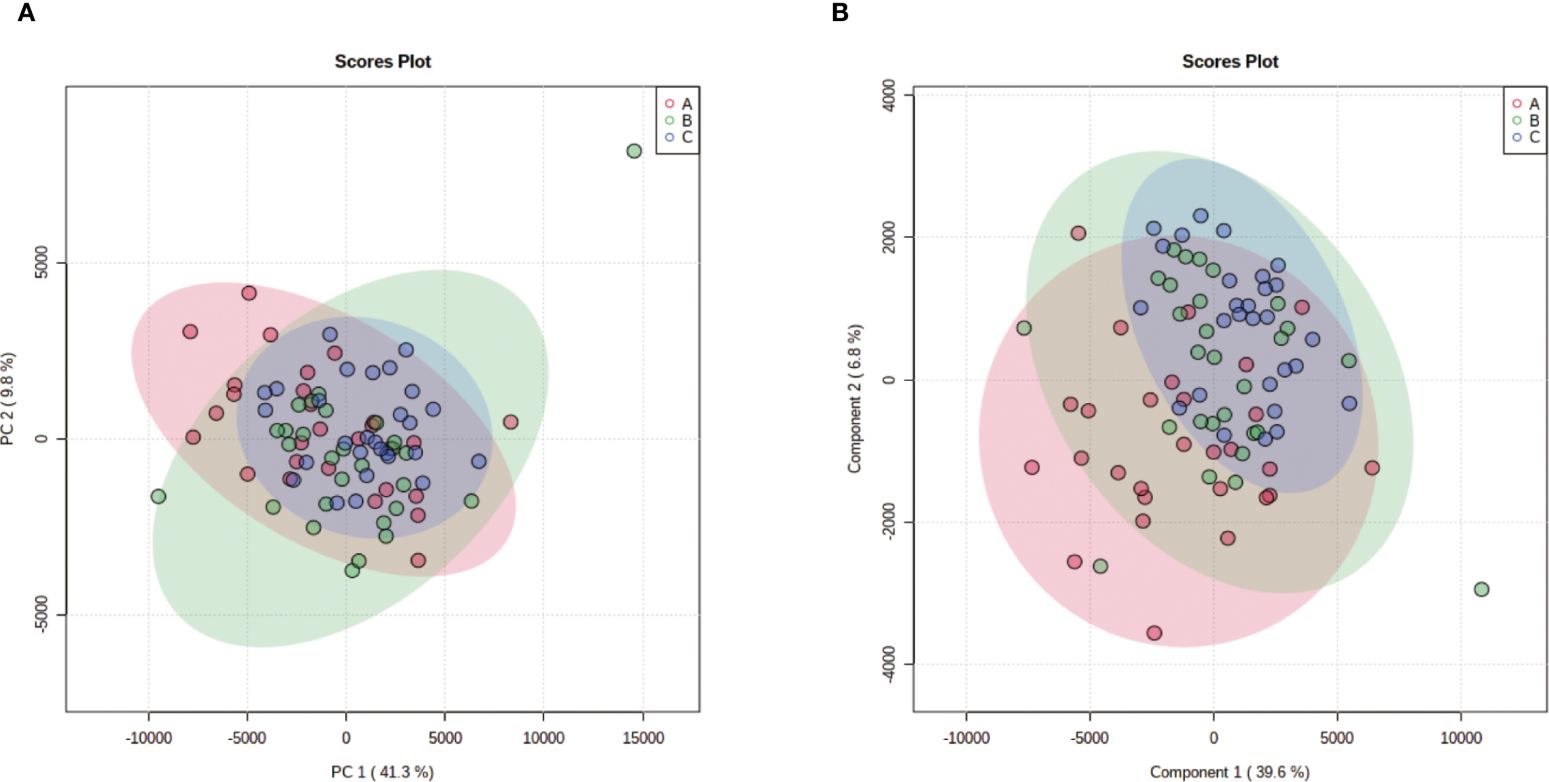
Multivariate statistical analysis of metabolic profiles. **(a)** Principal component analysis (PCA) score plot showing the intrinsic clustering and distribution of samples based on their metabolic profiles. The ellipses represent the 95% confidence interval for each group. **(b)** Partial least squares-discriminant analysis (PLS-DA) score plot demonstrating the separation between groups using a supervised method. The model was validated by permutation testing (n=9999) to guard against overfitting. Both plots indicate metabolic distinctions among the groups: A (Placebo, red), B (Treatment), C (Blank Control).

Unsupervised hierarchical clustering of the 27 dysregulated metabolites revealed distinct metabolic profiles that effectively separated ovarian endometriosis patients from controls. Notably, a coherent subcluster of steroid metabolites, including 17α-hydroxyprogesterone, showed a treatment-mediated reversal (**Figure 3A**: placebo group; B: treatment group; C: blank control group;The following pictures are all in this group). Principal component analysis (PCA) demonstrated significant group dispersion along PC1 (**Figure 4a**), with the treatment group clustered closer to controls than placebo (**Figure 4b**), indicating partial metabolic normalization.

**Figure 5:**
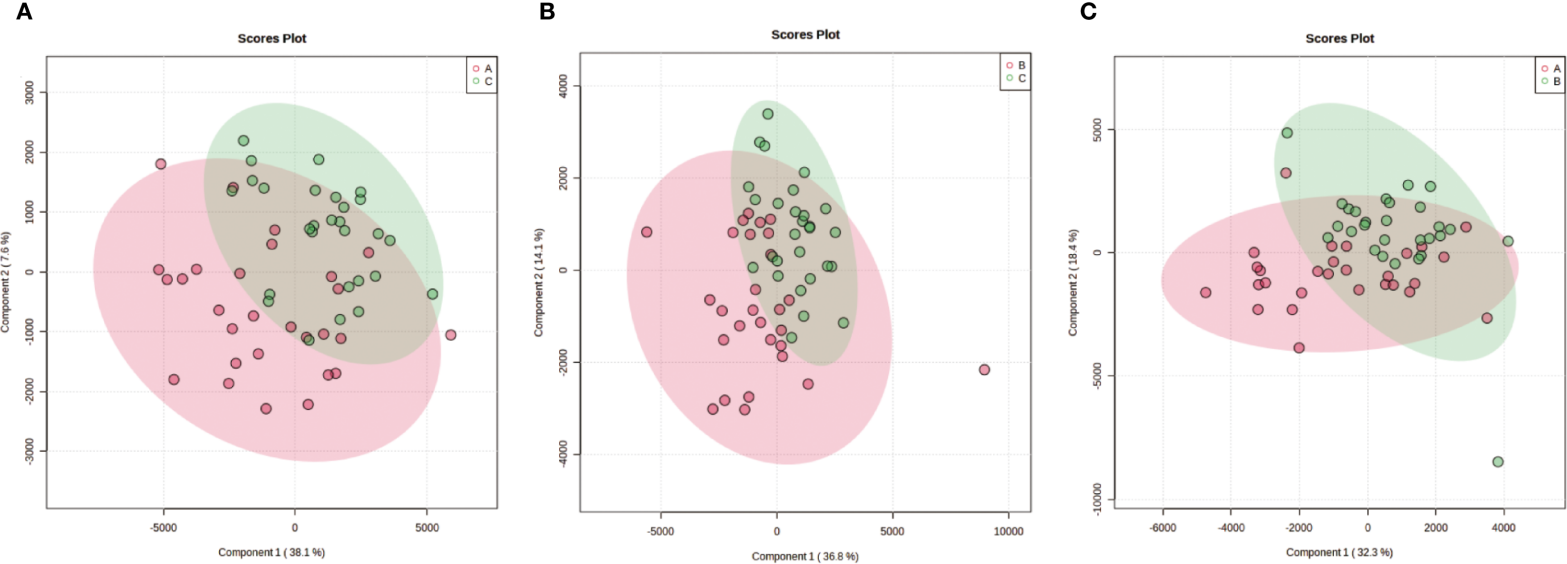
Pairwise partial least squares-discriminant analysis (PLS-DA) of metabolic profiles between groups. The analysis was performed using Metabolic Analyst to maximize separation and identify metabolic patterns distinguishing the groups. Score plots display clear separation between: **(a)** Group A (Placebo) and Group C (Blank Control); **(b)** Group C (Blank Control) and Group B (Treatment); **(c)** Group A (Placebo) and Group B (Treatment).

**Figure 6:**
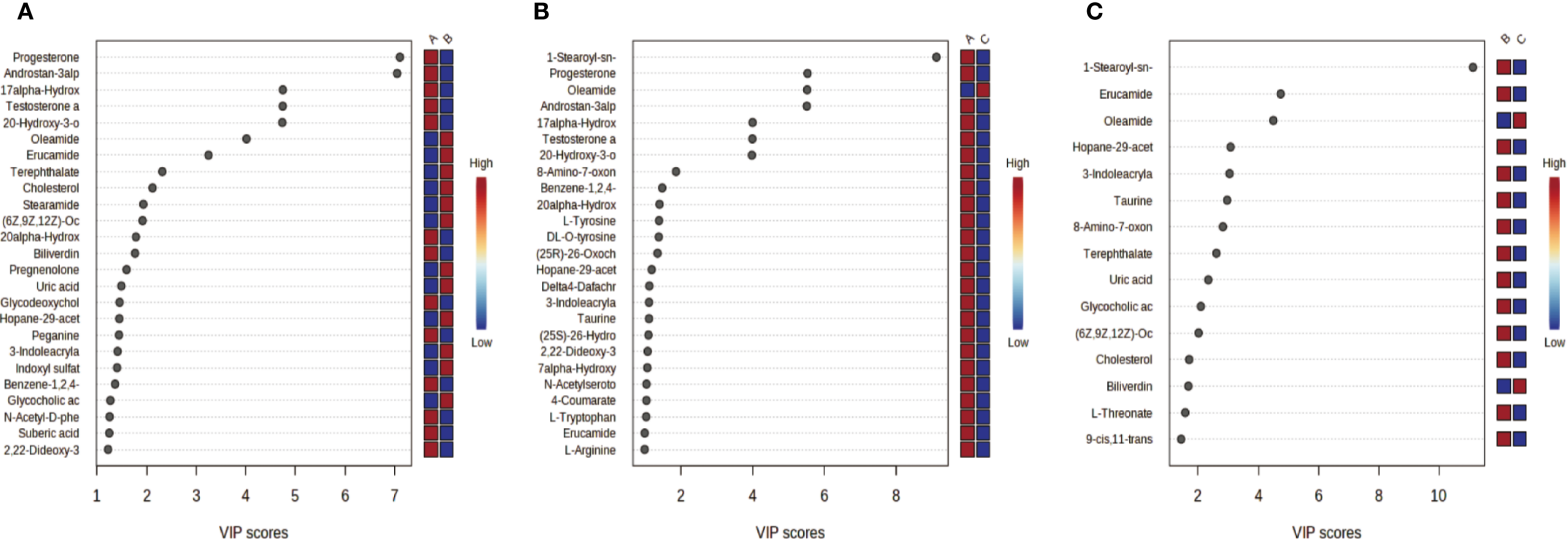
VIP Plots from PLS-DA. Variable importance in projection (VIP) plots derived from partial least squares-discriminant analysis (PLS-DA) quantify each metabolites contribution to inter group sample separation. VIP > 1 indicates significant inter-group differences (higher VIP greater contribution). Color coding: red = higher metabolite abundance in the first-listed group; blue lower abundance. **(a)** Group A (Placebo) vs. Group B (Treatment); **(b)** Group A (Placebo) vs. Group C (Blank Control); **(c)** Group C (Blank Control) vs. Group B (Treatment).

Partial Least Squares-Discriminant Analysis (PLS-DA) was performed using MetaboAnalyst software, revealing a clear separation between the placebo group and the blank control group. Overall, the treatment group following AAT clustered more closely with the blank control group, signifying a positive therapeutic effect (**Figure 4**). The VIP value for each sample metabolite was determined by PLS-DA, quantifying its contribution to sample separation. VIP values greater than 1 were considered to exert a significant effect. The VIPs obtained from pairwise PLS-DA comparisons across the three groups (**Figure 5**) are shown in **Figure 6**. A total of 27 differential metabolites were found across the three groups, including 25 upregulated metabolites: 9-cis,11-trans-octadecadienoate (6Z,9Z,12Z)-Octadecatrienoic acid, hopane-29-acetate, Erucamide, 4-(3-hydroxy-2-naphthyl)-2-oxobut-3-enoic acid, L-threonate, 4-coumarate, glycerophosphocholine8-amino-7-oxononanoateglycocholic acid (25R)-26-oxocholest-4-en-3-one, (25S)-26-hydroxycholest-4-en-3-one, 17alpha-hydroxyprogesterone, 1-stearoyl-sn-glycerol 3-phosphocholine2,22-dideoxy-3-dehydroecdysone20alpha-hydroxy-4-pregnen-3-one, 20-hydroxy-3-oxopregn-4-en-21-al, 7alpha-hydroxy-3-oxo-4-cholestenoatedelta4-dafachronic acid, DL-O-tyrosineL-arginineL-tyrosinetestosterone acetateglycodeoxycholic acid, and biliverdin. In contrast, two downregulated metabolites were found: dammara-20,24-diene and oleamide (**Tables 3** and **4**).

**Table 3:** List of different substances between the control and blank control groups.

Serial number	Chemical name	Ionization mode	Mass-to-charge ratio	Retention period	Characteristic fragment ions	Control content
1	(25R)-26-Oxocholest-4-en-3-one	[M+H]^+^	399.3243	13.16	184.0741; 264.2700	High
2	(25S)-26-Hydroxycholest-4-en-3-one	[M-H2O+H]^+^	383.3291	12.39	145.1031; 255.2121	High
3	17alpha-Hydroxyprogesterone	[M+H]^+^	331.2261	9.53	98.9848; 109.0648	High
4	1-Stearoyl-sn-glycerol 3-phosphocholine	[M+H]^+^	524.3704	12.50	104.1067; 184.0732; 258.1114; 341.3055; 506.3607	High
5	2,22-Dideoxy-3-dehydroecdysone	[M-H2O+H]^+^	413.3052	12.35	97.0663; 109.0651; 299.2385	High
6	20alpha-Hydroxy-4-pregnen-3-one	[M-H2O+H]^+^	299.2362	11.06	73.0465; 209.0296; 250.9899; 267.0187	High
7	20-Hydroxy-3-oxopregn-4-en-21-al	[M+H]^+^	331.2259	8.46	98.9848; 151.0749; 313.2162	High
8	4-Coumarate	[M+H]^+^	165.0541	1.41	65.0383; 77.0389; 95.0491; 123.0440	High
9	7alpha-Hydroxy-3-oxo-4-cholestenoate	[M-H2O+H]^+^	413.3033	11.44	97.0656; 147.1172	High
10	8-Amino-7-oxononanoate	[M-H]^-^	186.1130	5.97	57.0351; 97.0655; 125.0973	High
11	Delta4-Dafachronic acid	[M-H2O+H]^+^	397.3102	12.02	147.1174; 184.0748; 271.2078; 283.2409	High
12	DL-O-tyrosine	[M+H]^+^	182.0807	1.42	77.0378; 91.0543; 119.0490; 123.0442	High
13	L-Arginine	[M+H]^+^	175.1183	0.78	60.0557; 70.0652; 116.0709; 130.0974	High
14	L-Tyrosine	[M+H]^+^	182.0805	1.37	91.0547; 119.0498; 123.0441; 136.0757	High
15	Oleamide	[M+H]^+^	282.2789	13.75	69.0700; 83.0858; 121.1018; 247.2433	Low
16	Testosterone acetate	[M+H]^+^	331.2264	9.85	97.0647; 109.0650; 123.0808; 271.2063; 295.2062	High

**Table 4:** List of substances that differed between the control and treatment groups.

Serial number	Chemical name	Ionization mode	Mass-to-charge ratio	Retention period	Characteristic fragment ions	Treatment group content
1	(25S)-26-Hydroxycholest-4-en-3-one	[M-H2O+H]^+^	383.3291	12.39	145.1031; 255.2121	Low
2	17alpha-Hydroxyprogesterone	[M+H]^+^	331.2261	9.53	98.9848; 109.0648	Low
3	20alpha-Hydroxy-4-pregnen-3-one	[M-H2O+H]^+^	299.2362	11.06	73.0465; 209.0296; 250.9899; 267.0187	Low
4	20-Hydroxy-3-oxopregn-4-en-21-al	[M+H]^+^	331.2259	8.46	98.9848; 151.0749; 313.2162	Low
5	Glycodeoxycholic acid	[M+H]^+^	450.3213	10.88	414.2996; 321.2568; 158.0820	Low
6	Biliverdin	[M+H]^+^	583.2532	6.85		Low
7	Testosterone acetate	[M+H]^+^	331.2264	9.85	97.0647; 109.0650; 123.0808; 271.2063; 295.2062	Low

We performed Pearson correlation analysis using the relative quantitation values (chromatographic peak areas) of significantly altered metabolites and key clinical outcomes, including the total number of eggs and the total number of normally fertilized eggs. The results are summarized in **Supplementary Table S4**. Several metabolites, including 17α-Hydroxyprogesterone, (25S)-26-Hydroxycholest-4-en-3-one, and (25R)-26-Oxocholest-4-en-3-one, were correlated with the number of oocytes retrieved. After controlling for age, antral follicle count, and other relevant variables, only 17α-Hydroxyprogesterone retained a correlative trend with the number of oocytes retrieved (*r* = -0.286, *P* = 0.012). Additionally, after adjusting for covariates, 17α-Hydroxyprogesterone exhibited significant positive correlations with (25S)-26-Hydroxycholest-4-en-3-one (*r* = 0.337, *P* = 0.03) and (25R)-26-Oxocholest-4-en-3-one (*r* = 0.258, *P* = 0.026).

### 3.4 Metabolic pathway analysis

**Figure 7:**
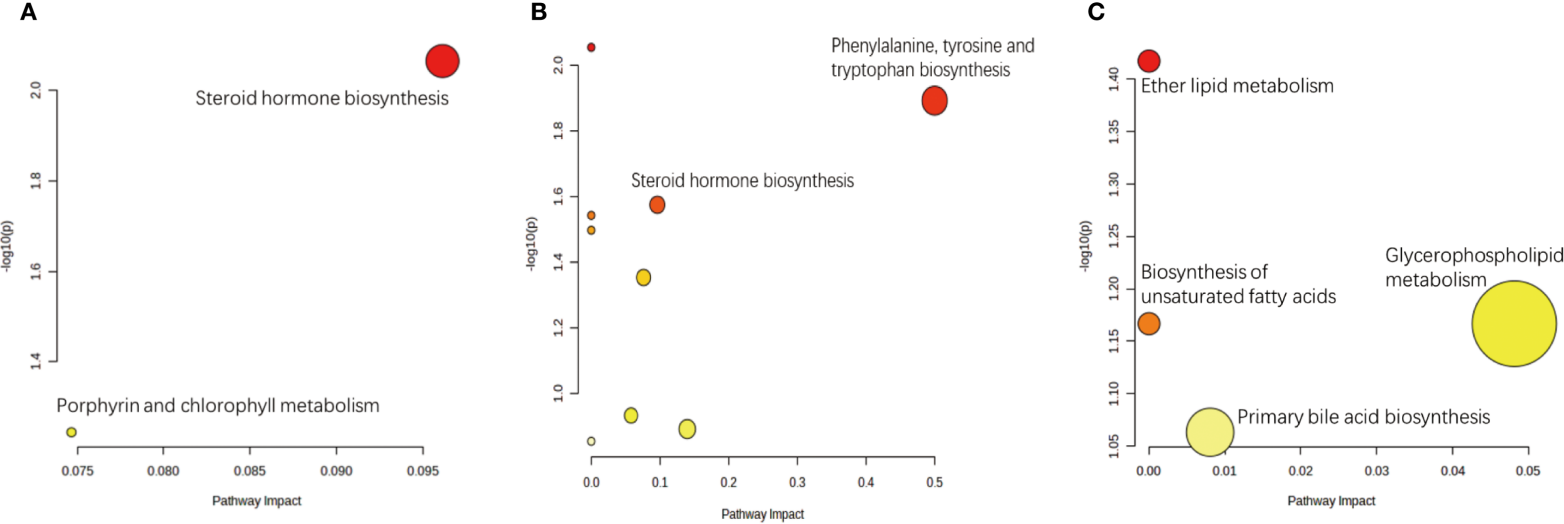
Pathway analysis of differential metabolites between comparison groups. Significantly enriched metabolic pathways were identified based on hypergeometric test and relative-betweenness centrality algorithms within the KEGG database. **(a)** The most relevant pathway distinguishing the Placebo group (A) and the Treatment group (B) was Steroid hormone biosynthesis. **(b)** Pathways significantly perturbed between the Placebo group (A) and the Blank Control group (C) included Phenylalanine, tyrosine and tryptophan biosynthesis and Steroid hormone biosynthesis. **(c)** The most relevant pathway differentiating the Treatment group (B) and the Blank Control group (C) was Ether lipid metabolism.

KEGG analysis allows for the investigation of gene function and genomic information. It offers a robust tool for metabolomics analysis and *in vivo* metabolic network research, assisting researchers in studying genes and their information as a complete network. This platform offers a comprehensive understanding of gene interactions and their effects. KEGG pathway analysis revealed a significant difference in the steroid hormone biosynthesis metabolic pathway between the treatment group and the placebo group. In addition, a significant difference in the ether lipid metabolism metabolic pathway was observed between the treatment group and the blank control group, as well as between the placebo group and the blank control group (**Figure 7**). The primary metabolic pathways linking the treatment group and the blank control group were associated with phenylalanine, tyrosine, and tryptophan biosynthesis, as well as steroid hormone biosynthesis. The above findings suggest that these pathways are involved in IVF in patients with EMs infertility who received the AAT. Overall, our findings collectively underscore the importance of the treatment in improving fertility.

## 4 Discussion

Our findings demonstrated that AAT could improve multiple IVF outcomes in OE patients, likely by restoring granulosa cell metabolic homeostasis. Ovarian endometriosis poses substantial burdens in ART, often necessitating additional pretreatment cycles and compromising ovarian stimulation by reducing Gn responsiveness evidenced by an increased Gn duration (10.62 ± 2.43 days vs. 9.00 ± 0.68 days in treatment, *P* < 0.05) and higher Gn dose (2549.04 ± 677.44 IU vs. 2112.50 ± 483.17 IU in treatment, *P* < 0.05), subjecting patients to disproportionate physical suffering (pain reduction Δ=-0.35 vs. Δ=-1.15 in placebo) and psychological distress (↓1.35 high-quality embryos vs. 3.04, *P* = 0.008) without commensurate outcomes. The significantly improved blastocyst development with AAT, demonstrated by increased fertilized oocytes (7.59 ± 4.58 vs. 4.46 ± 3.40, *P* = 0.001) and high-quality embryos (3.04 ± 1.89 vs. 1.35 ± 1.99, *P* = 0.008) compared to placebo, confirms its efficacy in mitigating ovarian dysfunction in endometriosis despite non-significant gains in oocyte retrieval (12.41 ± 7.27 vs. 8.85 ± 7.89, *P* = 0.12). Notably, the higher top-quality embryo rate in AAT-treated OE patients versus male-factor controls (47.02% vs. 29.05%, *P* = 0.023) must be interpreted with caution due to the “bucket-short effect” from uncontrolled semen limitations in the control group (supplementary semen parameters: progressive motility 17.7% ± 12.7% % vs 36.9% ± 13.8% in treatment group P<0.01). While this confounder precludes definitive conclusions about absolute embryo quality superiority, consistent enhancement of core embryological parameters, including a 41% reduction in Gn duration (*P* = 0.004) and 70% more transferable embryos (*P* = 0.005) over placebo, undeniably establishes its value for integrated OE management within active ART cycles by targeting granulosa cell metabolic dysregulation.

Metabolomics serves as a crucial tool for investigating disease progression and host metabolism (23). Recent advancements in metabolomics have established follicular fluid as a viable biomarker for objectively evaluating oocyte quality and predicting IVF-ET outcomes (24). In this study, untargeted metabolomic analysis of the three groups revealed significant differences in metabolites, leading to the identification of 27 key differentially abundant metabolites through KEGG pathway enrichment analysis. Comparison with databases such as Human Metabolome Database and Metlin revealed that these differential metabolites were predominantly enriched in lipids, organic acids, and organic heterocyclic compounds.

Clinical outcomes—including oocyte yield, fertilization rate, number of high-quality embryos, duration of Gn stimulation, and total Gn dose—were significantly poorer in the placebo group compared to both the AAT and blank control groups. These clinical observations suggest compromised ovarian response and oocyte quality in the placebo cohort. To investigate the molecular mechanisms underlying these findings, we performed KEGG pathway enrichment analysis, which identified steroid hormone biosynthesis as one of the most significantly altered metabolic pathways between the placebo group and the other two groups (**Figure 7**). Furthermore, correlation analysis between the relative concentrations of differential metabolites and clinical outcomes revealed a persistent linear relationship between 17α-hydroxyprogesterone and oocyte yield after controlling for other variables (*p* = 0.012, *r*=−0.286).

**Figure 8:**
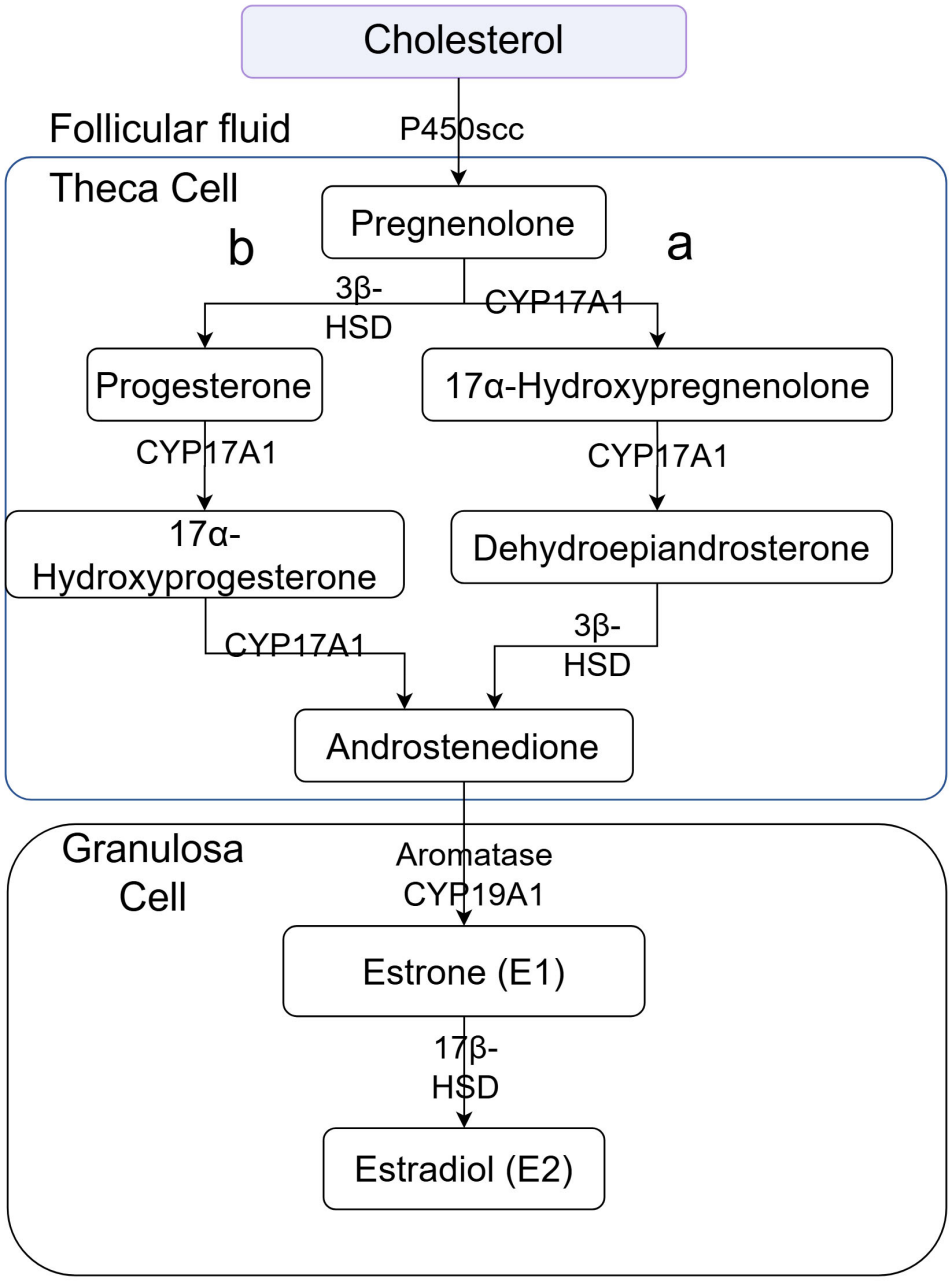
Illustration of the two-cell, two-gonadotropin theory for estrogen synthesis.

These results are consistent with the classic Two-Cell, Two-Gonadotropin Theory (**Figure 8**), in which theca and granulosa cells cooperate to produce ovarian steroids. Specifically, the placebo group exhibited marked elevations in 17α-hydroxyprogesterone (an intermediate of the Δ4 pathway), 20α-hydroxy-4-pregnen-3-one (a pregnenolone derivative associated with functional inactivation), and several testosterone analogs (**Tables 3**, **4**). The increase in 17α-hydroxyprogesterone suggests compensatory activation of the Δ4 pathway in theca cells (**Figure 2b**). Similarly, elevated levels of testosterone analogs—primarily synthesized in theca cells—reflect enhanced thecal steroidogenic activity. However, the absence of a concomitant rise in estrogen derivatives (**Figures 6a, b**) implies impaired aromatase-mediated conversion within granulosa cells. We propose two plausible, non-mutually exclusive explanations (1): granulosa cells in the placebo group may have reached their maximum steroidogenic capacity, or (2) a partial loss of aromatase activity in a subset of granulosa cells may have triggered compensatory substrate accumulation to sustain estradiol synthesis. We favor the latter hypothesis, as it is further supported by the pronounced accumulation of 20α-hydroxy-4-pregnen-3-one—a metabolite predominantly produced by luteinized granulosa cells. Thus, we speculate that premature luteinization of granulosa cells may underlie the aberrant steroid metabolism, eliciting compensatory responses from theca cells. It should be noted, however, that 20α-hydroxy-4-pregnen-3-one may also be synthesized at low levels by theca cells. Therefore, although our data strongly implicate luteinized granulosa cells, the exact cellular origin remains uncertain and warrants further investigation. 

Notably, the AAT treatment group exhibited a metabolic profile markedly distinct from the placebo group, with no significant alterations in steroidogenic intermediates observed, rendering it comparable to the blank control group (**Table 5**). These findings indicate that AAT effectively restores the functional dynamics of the follicular steroidogenic microenvironment. Mechanistically, AAT appears to mitigate premature luteinization of granulosa cells, thereby preserving their aromatase activity and capacity to convert testosterone precursors into estrogens. Concurrently, the intervention likely attenuates compensatory hyperactivity in theca cells, rebalancing the Δ4 and Δ5 steroidogenic pathways. By targeting these underlying cellular dysfunctions, AAT reestablishes steroidogenic homeostasis, which is fundamental to rescuing oocyte developmental competence and ultimately improving clinical outcomes in affected individuals.

**Table 5:** List of substances that differed between the blank control groups and treatment groups.

Serial number	Chemical name	Ionization mode	Mass-to-charge ratio	Retention period	Characteristic fragment ions	Treatment group content
1	cis,11-trans-Octadecadienoate	[M+NH4]+	298.2733	15.95	266.0242; 109.1007; 81.0700	High
2	(6Z,9Z,12Z)-Octadecatrienoic acid	[M+NH4]+	296.2575	11.23	251.1871; 117.0911; 59.0494	High
3	Hopane-29-acetate	[M+K]+	509.3779	11.94	240.9636; 184.0740	High
4	Erucamide	[M+H]+	338.3417	16.10	303.3072; 204.1610; 57.0701	High
5	4-(3-Hydroxy-2-naphthyl)-2-oxobut-3-enoic acid	[M-H2O+H]+	225.0604	10.94	192.9993; 164.9684	High
6	L-Threonate	[M+H]+	137.0454	3.55	119.0353; 95.0855; 81.0699	High
7	4-Coumarat	[M+H]+	165.0541	1.41	65.0383; 77.0389; 95.0491; 123.0440	High
8	Glycerophosphocholine	[M+H]+	258.1098	10.55	73.0640	High
9	8-Amino-7-oxononanoate	[M-H]-	186.1130	5.97	57.0351; 97.0655; 125.0973	High
10	Glycocholic acid	[M+H]+	466.3157	9.68	430.3001; 412.2874; 209.1345	High
11	Dammara-20,24-diene	[M+K]+	449.3606	12.42	327.2326	Low

Our metabolomic findings provide compelling, albeit indirect, evidence suggesting that AAT intervention may alleviate oxidative stress within the follicular microenvironment of OE patients. The elevated levels of oxidized cholesterol metabolites, namely (25R)-26-Oxocholest-4-en-3-one and (25S)-26-Hydroxycholest-4-en-3-one, in the placebo group compared to the blank control, directly indicate that steroidogenic precursors are subjected to reactive oxygen species (ROS) attack. The significant reduction of these oxidized metabolites in the AAT group points to a decrease in ROS-mediated damage. Furthermore, this improvement in the redox environment is supported by the elevated levels of antioxidative substrates—including 4-Coumarate and L-Threonate—observed in the treatment group (25, 26).

Notably, the reduction of biliverdin in the AAT group, though paradoxical given its role as an antioxidant (27), may reflect a lowered demand for enzymatic antioxidant defense due to improved redox status. However, given the complex regulation of biliverdin metabolism, this change could also be influenced by non-redox factors and should therefore be interpreted with caution. Beyond oxidative stress, the correlative patterns within our metabolic network suggest a complex, self-amplifying vicious cycle between oxidative stress and steroidogenic disruption. This is evidenced by several key correlations: oxidative stress metabolites showed a strong association with the luteinization marker 20α-hydroxy-4-pregnen-3-one (*P* = 0.001, *r=*0.43) and the steroidogenic intermediate 17α-hydroxyprogesterone (*P* = 0.005, *r=*0.318). The absence of a direct correlation between oxidative stress metabolites and the total number of eggs, coupled with the significant negative correlation between 17α-hydroxyprogesterone and oocyte yield (*r=*-0.286), further implies that oxidative stress may exert its detrimental effects indirectly through disrupting steroidogenic homeostasis. These correlative patterns support a bidirectional interplay: oxidative stress is known to inhibit key steroidogenic enzymes such as aromatase, leading to accumulation of precursors like 17α-hydroxyprogesterone. Conversely, intensified steroidogenic activity can generate reactive oxygen species as metabolic byproducts, thereby exacerbating oxidative stress. This cyclic interaction likely propagates granulosa cell dysfunction, promoting premature luteinization—as reflected by the pronounced rise in 20α-hydroxy-4-pregnen-3-one. The ability of AAT to concurrently mitigate oxidative damage and restore steroidogenic balance suggests that it may disrupt this pathogenic cycle at multiple points. Future studies employing targeted oxidative stress assays, mediation analysis, and longitudinal sampling are warranted to dissect the temporal hierarchy and causal pathways underlying this aberrant metabolic crosstalk and to definitively establish the antioxidant role of AAT in the follicular microenvironment.

Beyond oxidative stress, our metabolomic analysis of follicular fluid revealed a broader spectrum of metabolic alterations following AAT treatment, underscoring the multifaceted nature of OE pathophysiology. The distinct metabolic profiles observed—particularly the elevated levels of amino acid synthetic metabolites in the blank control group—may reflect enhanced protein synthesis rates within the follicle, suggesting a potentially more active or supportive follicular microenvironment in the absence of disease and intervention (28). In contrast, the accumulation of diverse organic acids in the placebo group may be indicative of aberrant peroxidation and disrupted energy metabolism associated with OE. The significant alteration in oleamide levels across groups merits careful interpretation. Existing literature presents conflicting evidence regarding its bioactivity: oleamide has been reported to exert both pro- and anti-proliferative effects in different cellular contexts, while also demonstrating anti-inflammatory and antioxidant properties (29–32). This ambiguity highlights the context-dependent nature of oleamide signaling and underscores the need for further investigation to clarify its specific role in human follicular fluid. Collectively, these modulated pathways imply that AAT may exert therapeutic benefits not through a single mechanism, but via a coordinated interaction with multiple metabolic processes—including protein synthesis, energy metabolism, redox balance, and inflammatory signaling—ultimately contributing to an improved follicular milieu in OE.

Furthermore, comparative metabolomic profiling identified seven key metabolites whose expression shifted from upregulation to downregulation following AAT intervention, suggesting their potential as therapeutic biomarkers in the follicular fluid of OE patients. These metabolites include (25S)-26-hydroxycholest-4-en-3-one (27-HC), 17α-hydroxyprogesterone (17α-OHP), 20α-hydroxy-4-pregnen-3-one, 20-hydroxy-3-oxopregn-4-en-21-al, (25R)-26-Oxocholest-4-en-3-one, biliverdin, and testosterone acetate. Their altered levels may reflect modulation of steroidogenesis and redox homeostasis in response to AAT, positioning them as promising candidates for monitoring treatment efficacy in ovarian endometriosis.

The design of our acupoint application therapy (AAT) protocol, encompassing both the specific acupoints and the customized herbal formulation, was predicated on the principles of Traditional Chinese Medicine (TCM) and their potential mechanistic relevance to the pathophysiology of ovarian endometriosis (OE). The selection of Shenque (CV8) is of particular significance. Located at the umbilicus, this point is considered a pivotal hub for regulating the Ren and Chong meridians, which are fundamentally associated with uterine and ovarian function in TCM theory. Its thin subcutaneous layer and high vascularity also make it an ideal portal for systemic transdermal drug delivery. The application of a warming and blood-activating herbal mixture comprising Zingiber officinale (Ganjiang) and Chuanxiong rhizoma (Chuanxiong) at this site was intended to counteract the cold and stagnant blood stasis pattern often associated with OE (41), potentially improving local pelvic microcirculation (42) and mitigating the inflammatory microenvironment (43). The inclusion of bilateral Yongquan (KI1), the first point on the Kidney meridian, was primarily intended to tonify Kidney essence—a fundamental concept in TCM regarded as the root of reproductive health. According to TCM theory, insufficient Kidney essence not only contributes to infertility but is also closely associated with sleep disturbances, anxiety, and emotional imbalance, which are common comorbidities in OE patients. The herbs applied at this site—Evodia ruteacarpa (Wuzhuyu), which exhibits anti-inflammatory and calming properties, and borneol (Bingpian), which enhances transdermal penetration—were selected not only to regulate endocrine-immune axis dysfunction but also to promote emotional stability and improve sleep quality, thereby addressing both systemic metabolic dysregulation and psychological stress. Simultaneously, Sinapis alba (Baijiezi) and Asarum sieboldii (Xixin) were applied at bilateral Yashi points to reduce local swelling and alleviate pain (43,44). By mitigating physical discomfort and inflammation, this combination may further contribute to improved sleep (45,46) and overall well-being, forming a holistic therapeutic strategy that targets both physiological and psychological aspects of OE.

We hypothesize that the therapeutic effect of AAT is achieved through a synergistic, multi-target mechanism. The physical stimulation of the acupoints may modulate neuroendocrine signaling and optimize the body’s internal milieu for receptivity to treatment. Concurrently, the transdermally delivered bioactive compounds directly target key pathological processes: reducing inflammation (e.g., via Wuzhuyu, Xixin), alleviating oxidative stress, resolving stasis (e.g., via Chuanxiong), and improving microcirculation. This combined “point-drug” approach may collectively ameliorate the follicular microenvironment, rescue granulosa cell steroidogenic function, and ultimately support improved oocyte development and IVF outcomes. Future studies employing separate arms for acupoint stimulation alone and herb application alone are warranted to dissect their individual contributions to the observed clinical benefits.

In summary, this preliminary investigation suggests that acupoint application therapy (AAT) may offer potential for improving IVF outcomes in patients with ovarian endometriosis (OE). Untargeted metabolomic profiling of follicular fluid revealed distinct metabolic alterations in OE patients, indicating that AAT might alleviate oxidative stress in granulosa cells and restore steroidogenic function. These findings provide a theoretical basis for incorporating traditional Chinese medicine into clinical management strategies. However, this study has several limitations, including a limited sample size and an observational design that cannot establish causality. Furthermore, the initial experimental design did not permit full elucidation of the biological crosstalk between oxidative stress and steroid metabolic dysregulation in OE. To address these gaps, future research should employ more foundational experimental models, including *in vitro* cell cultures and animal studies, to explicitly investigate the mechanistic actions of AAT and its active components. Such approaches could help clarify the pathways involved in granulosa cell function and steroid hormone synthesis, and provide deeper insights into the intervention’s mode of action. Validation through well-controlled preclinical studies will be essential to confirm these preliminary observations and support the translation of AAT into clinical assisted reproductive protocols for OE.

## Data Availability

The original contributions presented in the study are included in the article/**Supplementary Material**. Further inquiries can be directed to the corresponding author/s.
