# Supplementary material for: Acupoint application improves IVF outcomes and rescues granulosa cell steroid metabolic dysregulation in ovarian endometriosis
Source: Front Endocrinol (Lausanne). 2025 Oct 27;16:1665669. doi: 10.3389/fendo.2025.1665669 (PMC12597724; doi:10.3389/fendo.2025.1665669)
Supplement: Supplementary file 1 [file Table1.docx]

Table S1 The basic information of the samples sent for testing

| Sample Grouping | Sample size | Sample Status |
| --- | --- | --- |
| Treatment group | 27 | Liquid |
| Placebo group | 26 | Liquid |
| No-treatment control | 28 | Liquid |

Table S2 Chromatographic analysis

| Time (min) | organic phase B (%) |
| --- | --- |
| 0 | 1 |
| 1.5 | 1 |
| 13 | 99 |
| 16.5 | 99 |
| 16.6 | 1 |
| 20 | 1 |

Table S3. Difference in transient abdominal pain scores before and after intervention [（Δ
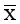
±s），%]

| Syndrome | Treatment group (n=27) | Placebo group (n=26) | *T* | *P* |
| --- | --- | --- | --- | --- |
| Abdominal pain before or after menstruationAbdominal Pain | -1.15±0.36 | -0.35±0.49 | 6.84 | 0.01 |

Table S4. Statistical analysis of clinical outcomes and differential metabolites (Pearson)

| Pearson correlation coefficient | 4-Coumarate, | 17α-Hydroxyprogesterone | 20α-Hydroxy-4-pregnen-3-one | (25S)-26-Hydroxycholest-4-en-3-one, | (25R)-26-Oxocholest-4-en-3-one | L-Threonate | Total number of eggs | Total number of normally fertilized eggs |
| --- | --- | --- | --- | --- | --- | --- | --- | --- |
| 4-Coumarate, | 1 |  |  |  |  |  |  |  |
| 17α-Hydroxyprogesterone | 0.074 | 1 |  |  |  |  |  |  |
| 20α-Hydroxy-4-pregnen-3-one | 0.119 | 0.186 | 1 |  |  |  |  |  |
| (25S)-26-Hydroxycholest-4-en-3-one, | 0.169 | .486^**^ | .474^**^ | 1 |  |  |  |  |
| (25R)-26-Oxocholest-4-en-3-one | 0.209 | .521^**^ | 0.201 | .490^**^ | 1 |  |  |  |
| L-Threonate | .485^**^ | 0.123 | 0.099 | 0.075 | .318^**^ | 1 |  |  |
| Total number of eggs | -0.133 | -.563^**^ | -0.120 | -.253^*^ | -.343^**^ | -0.039 | 1 |  |
| Total number of normally fertilized eggs | -0.188 | -.505^**^ | -0.090 | -0.197 | -.344^**^ | -0.143 | .857^**^ | 1 |

Table is a Pearson correlation analysis, not controlling for other variables，The values in the table are Pearson Correlation Coefficient
**. Significant correlation at the 0.01 level (two-tailed)
*. Significant at the 0.05 level (two-tailed).
